# Supplementary material for: The South American MicroBiome Archive (saMBA): enriching the microbiome field by studying neglected populations
Source: Nat Commun. 2025 Aug 9;16:7371. doi: 10.1038/s41467-025-62601-4 (PMC12335589; doi:10.1038/s41467-025-62601-4)
Supplement: Supplementary file 1 — Supplementary Information [file 41467_2025_62601_MOESM1_ESM.pdf]

The South American MicroBiome Archive (saMBA): Enriching the Microbiome Field by Studying Neglected Populations

Benjamin Valderrama[1,2,*], Paulina Calderon-Romero[3], Thomaz F.S. Bastiaanssen[4], Aonghus Lavelle[1,2], Gerard Clarke[1,5], John F Cryan[1,2,*]

Affiliations

1. APC Microbiome Ireland, Cork, Ireland
2. Department of Anatomy and Neuroscience, University College Cork, Cork, Ireland
3. Center for Integrative Biology, Faculty of Sciences, Universidad Mayor, Santiago, Chile
4. Department of Psychiatry, Amsterdam University Medical Centers Location Vrije Universiteit Amsterdam, the Netherlands
5. Department of Psychiatry & Neurobehavioural Sciences, University College Cork, Cork, Ireland

* Co-corresponding authors

World regions used in previous work are too broad, justifying the analysis of gut microbiomes in narrower areas

The HMC [1], a previous work performed an automated search of gut microbiome samples from humans across the different world regions defined by the United Nations. Considering the diverse lifestyles within the 'Latin America and the Caribbean' region, we hypothesised that the gut microbiome of people living in Central and South America would be substantially different, potentially justifying narrower efforts to better characterise the subpopulations. A re-analysis of the HMC resource shows differences in the gut microbiomes of people in those subregions. While the abundance of Bacillota and Methanobacteriota are higher in subjects from Central America, South Americans show higher Bacteroidota and Pseudomonadota. This result suggests that explorations at a finer geographic resolution are justified.

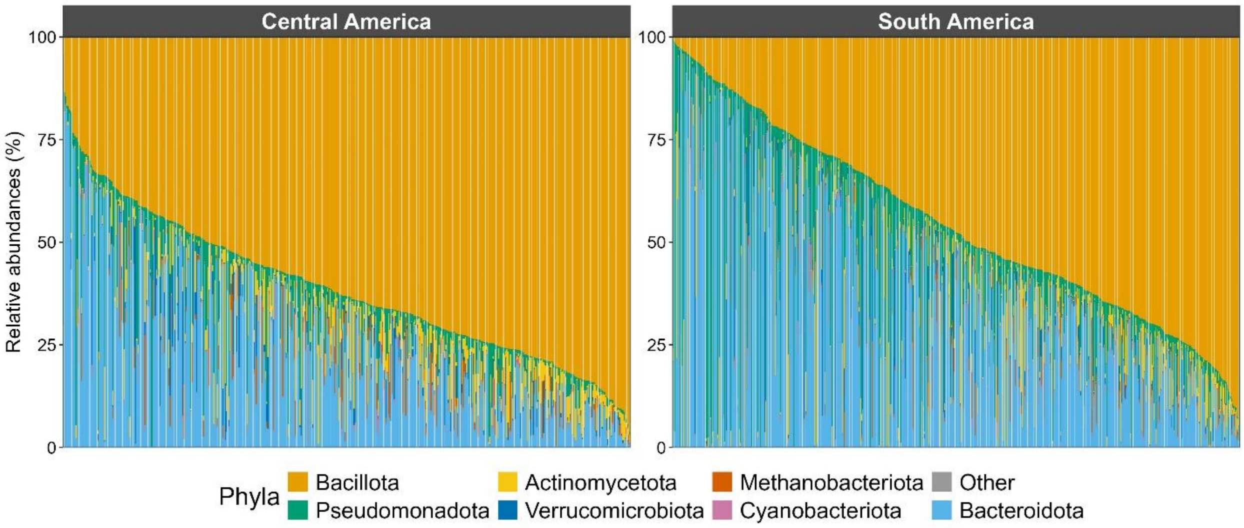

Supplementary Figure 1: Microbiome samples from Central and South America show different compositions, justifying an independent exploration of each region. Panel shows the relative abundance of the most abundant phyla identified in Central America (left panel) and South America (right panel). Each colour depicts one of the 7 most abundant phyla across Latin America and the Caribbean. Grey represents the relative abundance of all other phyla. Samples in each panel are ordered by their relative abundance of the phylum Bacillota.

Discarding projects with less than 50 samples likely exacerbates existing differences in sampling efforts across world regions

In the HMC study, projects with less than 50 sequenced samples were excluded when building the resource [1]. We hypothesised that this criterion may potentially impact world regions in a way that exacerbates existing differences in sampling efforts, as poorer regions may have higher proportion of projects with less than 50 sequenced samples. While a direct assessment of this impact would require detailed manual curation of microbiome studies (as described in our methods), we believe that a reanalysis of the HMC data can still offer valuable insights.

We examined the distribution of the number of microbiome samples per project across world regions and calculated the global median (Supplementary Figure 2A). We then determined the proportion of projects in each region that exceeded this global median. In some regions—such as 'Sub-Saharan Africa' (~58%), 'Europe and North America' (~54%), and 'Central and Southern Asia' (~54%)—approximately half or more of the projects have sample sizes above the global median. In contrast, 'Latin America and the Caribbean' show the lowest proportion of projects exceeding the global median sample size (~22%). This suggesting a particularly pronounced vulnerability to the exclusion criteria used in the HMC, as most sequencing projects in this world region are smaller than the rest of the globe.

Additionally, we conducted a similar analysis at the country level, in which we assessed the number of microbiome samples per country across the world and calculated the global median (Supplementary Figure 2B). We then determined the proportion of countries within each region that exceeded this global median. Note that although included in the analysis, China and the United States were excluded from the plot due to sample counts that are order of magnitudes higher than the next most-sampled country. In this case, the world regions with more countries exceeding the global median are 'Australia and New Zealand' (100%), 'Europe and North America' (~68%) and 'Eastern and South-Eastern Asia' and 'Central and Southern Asia' (both with ~66%). Strikingly, 'Latin America and the Caribbean' was the only region where all countries had fewer samples than the global median.

Although we initially hypothesised that excluding microbiome studies by the number of samples could exacerbate global underrepresentation, our reanalysis of the HMC data suggests that this may not be a concern for all regions with a high proportion of Low- and Middle-Income Countries. Regardless, our results strongly suggest that the decision of excluding microbiome studies by the number of samples affects 'Latin America and the Caribbean' the most. This result further underscores the relevance of saMBA, a resource that expands our understanding of gut microbiome diversity in a highly biodiverse yet profoundly understudied world region.

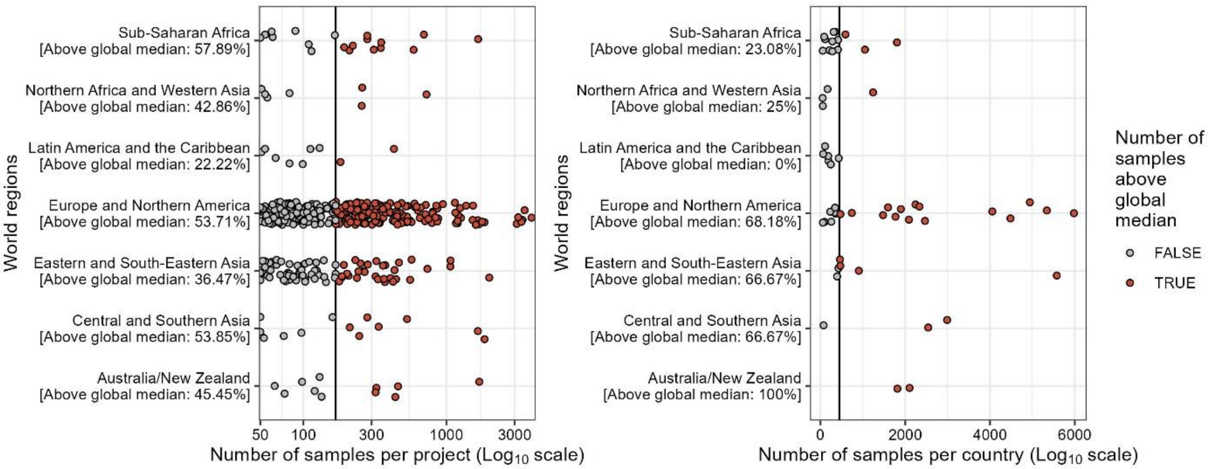

Supplementary Figure 2: Global distributions of the number of samples per microbiome sequencing project and per country. (A) Number of samples per project within each world region defined in the HMC. (B) Number of samples per country within each world region defined in the HMC. In both panels, the vertical black line represents the global median. Grey dots represent projects (in panel A) and countries (in panel B) with fewer samples than the global median. Red dots represent the opposite. For each world region, we calculated the proportion of projects (in panel A) and countries (in panel B) that are above the global median.

Schematic of the workflow used to build saMBA improves transparency in the bioinformatics analysis of samples

To improve transparency in our work, we show the workflow deployed in the analysis of each project included in saMBA. Each step was described in the methods and implemented as described in the GitHub repositories linked on each methods section. As a complement, the following schematic illustrates the steps of the workflow in a visual manner. Along with each step of the workflow (see the first column), we provide a small description of it (second column) and the number of samples successfully completing it (third column). A total of 3,382 human faecal samples from 9 South American countries were screened. After applying the quality filters described in Supplementary Figure 3, a total of 2,913 samples from the 9 countries were finally included in the resource.

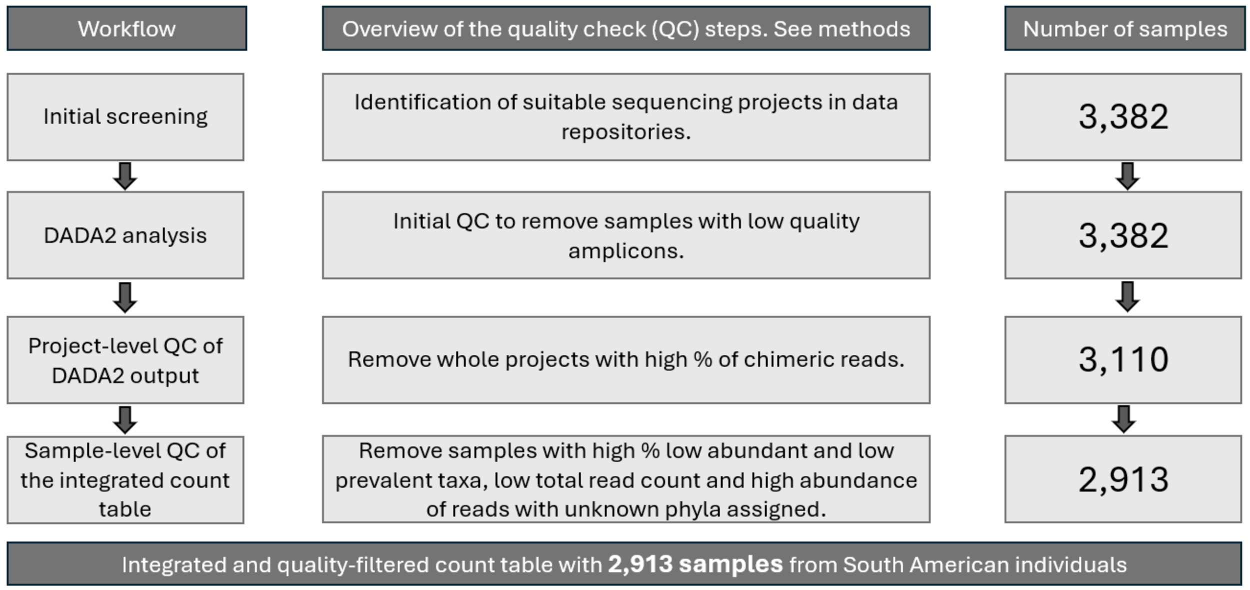

Supplementary Figure 3: Number of samples successfully completing each step in the analysis workflow. Each step in the workflow has a brief explanation that matches the information available in the methods section.

The community profiling of samples analysed with saMBA and the HMC workflows are concordant.

It is known that different processing workflows can generate slightly different results when applied to analyse the same dataset. Since the workflow developed to build the HMC was not available for public reuse, we aim to recreate the workflow following the details provided by the authors in the method section and the non-reusable code shared on an archived GitHub repository [1]. The goal was to replicate the workflow as closely as possible. To examine how well we matched their processing steps, we followed qualitative and quantitative approaches. First, we compared the relative abundances of the seven most abundant phyla in samples included in both workflows (Supplementary Figure 3A). Although Actinomycetota was more abundant in some samples analysed with the saMBA workflow when compared to the same sample analysed with the HMC workflow, the overall relative abundances are concordant for most samples.

Next, we quantified the dissimilarity between samples analysed with each workflow to determine how different are the results generated by each of them when analysing the same sample. If the same sample is analysed with different workflow and they are highly dissimilar, it implies that the source of that dissimilarity is technical, as it comes from small differences in the bioinformatics analysis. However, if different samples are analysed with the same workflow and they are highly dissimilar, then the source is biological, not technical. Thus, one indication that workflow results are compatible is that the average dissimilarity resulting from analysing the same sample with different workflows is lower than the average dissimilarity of different samples analysed with the same workflow. In other words, if the biological sources of dissimilarity are on average higher than the technical sources. This analysis was done at the genus level (Supplementary Figure 3B).

To provide a reference, we added two other groups, where we calculated the dissimilarity of different samples analysed with different workflow (i.e., technical and biological source of dissimilarity), and the same sample analysed with the same workflow (i.e., no dissimilarity). Although some samples analysed with different workflows show high levels of dissimilarity, the boxplots show that more than 75% of the data in that group show less dissimilarity than any different samples analysed with the same workflow. Additionally, approximately 25% of the data shows no dissimilarity. This suggests that in most cases, the results generated by the workflow used to build the HMC and saMBA generate compatible results (Supplementary Figure 3B).

To further evaluate the similarity between distance matrices obtained from our implementation of the HMC workflow, we performed a Mantel test using Bray-Curtis dissimilarities calculated at the genus level. This test was used to assess whether pairwise relationships between samples are preserved across workflows. High correlation values indicate that biological sources of variation (i.e., differences between samples) captured

by both workflows agree, whereas low correlation values would suggest that technical differences between workflows may obscure the biological sources of difference.

The Mantel statistic (r = 0.9813, p = 9.99 × 10^{-5}, 10,000 permutations) indicated a very strong and statistically significant correlation between the two matrices. This result confirms that our workflow preserves the core structure of microbial community dissimilarities observed in the HMC workflow. Consequently, it supports the compatibility of the two workflows, suggesting that results from saMBA can be integrated with those from the HMC workflow without additional normalization or preprocessing.

Finally, we sought to explore the overall dissimilarities between workflows at the genus level (Supplementary Figure 3C). The ordination plot shows that although there is variability across samples in the first two dimensions, there is considerable overlap in the space used by samples analysed with different workflows. This is consistent with the results shown in Supplementary Figure 3B.

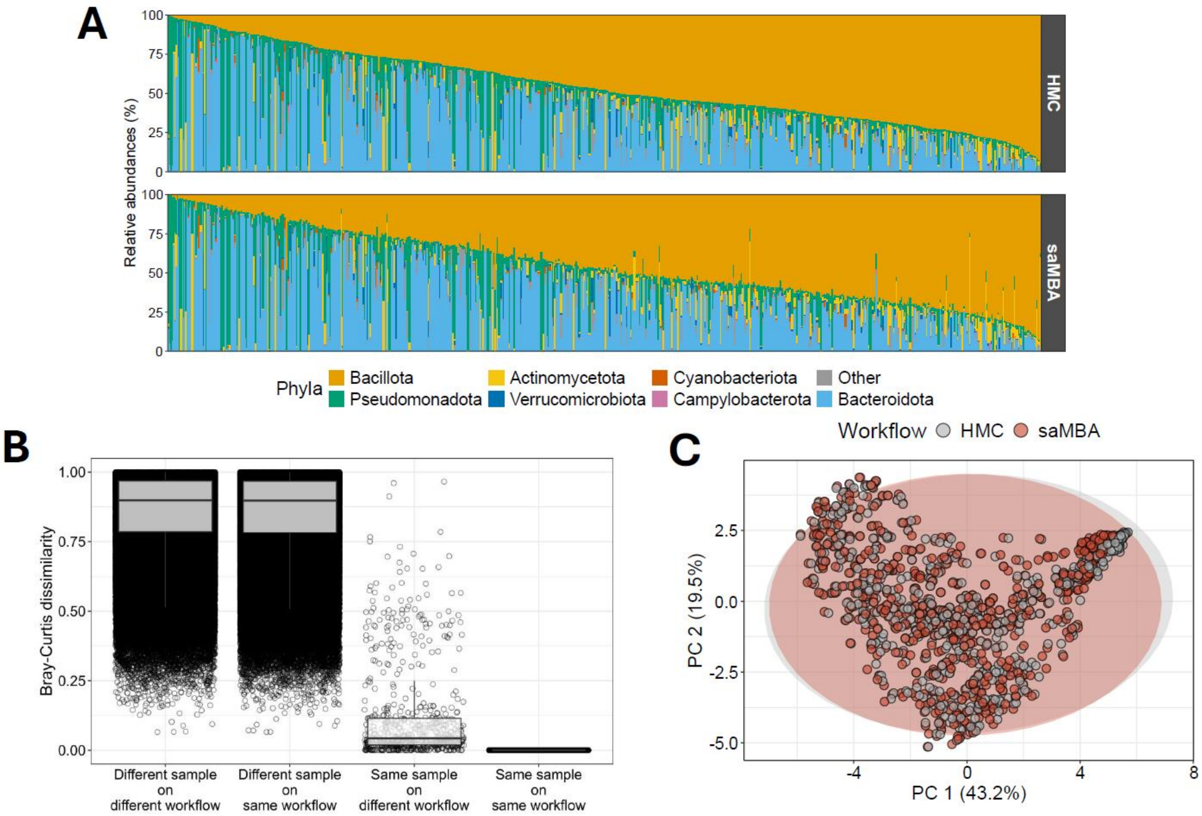

Supplementary Figure 4: Qualitative and quantitative assessment of the dissimilarity between results generated by the HMC and saMBA workflows. (A) Relative abundances at the phylum-level in samples included in the HMC (top) and in saMBA (bottom). Each colour depicts one of the 7 most abundant phyla, and grey represents the relative abundance of all other phyla. (B) Boxplot of the Bray-Curtis dissimilarities between pair of samples at the genus level. Pairs are either (1) different

samples analysed with different workflows, (2) different samples analysed with the same workflow, (3) the same sample analysed with different workflows and (4) same sample analysed with the same workflow. (C) PCoA plot of using Bray-Curtis dissimilarities at the genus level. Orange depicts samples analysed with the HMC workflow, and blue those analysed with the saMBA workflow.

Diversity estimates across the region are not altered by uneven sampling depths among countries.

The subsampling process (see methods) was conducted across all countries to estimate their biodiversity, as well as the continental biodiversity (Figure 3A, Supplementary Figure 5). Sequencing depths of projects included in saMBA may not be evenly distributed across countries. Thus, a fair comparison of the biodiversity between different countries may require accounting for the disparate sequencing depth.

Consequently, we repeated the analysis conducted in samples without rarefaction (Figure 3A and Supplementary Figure 5A and 5D). This time, we added an extra step where samples were rarefied to either 9,000 reads (Supplementary Figure 5B and 5E) or 1,000 reads (Supplementary Figure 5C and 5F) before estimating the number of unique taxa among all samples included on each iteration. The selected number of reads represent 90% and 10% of the total of reads found in the sample with the lowest number of reads, respectively. We repeated the same process for the saMBA-wide count table before and after applying the filtering protocol described in the methods section, under the title 'Building saMBA'.

Note that rarefying to 9,000 reads (Supplementary Figure 5B and 5E) marginally reduce the number of unique taxonomic entries in Venezuela (orange) and Brazil (pale purple) and Peru (blue), when compared to the non-rarefied curves (Supplementary Figure 5A and 5D). Other countries, as well as the continental estimate (black line), remain almost unaltered. On the other hand, rarefying to 1,000 reads (Supplementary Figure 5C and 5F) significantly reduces the estimated richness in all countries, as well as the continent estimate (black line), when compared to the non-rarefied curves (Supplementary Figure 5A and 5D).

Note that regardless of the rarefaction regimen used, the order of countries (from most to less biodiverse) remained almost the same when comparing them to the non-rarefied curves. This suggests that unequal sampling efforts across countries are probably not severely distorting the estimates of the country or continental biodiversity, probably due to the scale of the analysis.

In contrast, the effect of filtering out rare taxa and samples from saMBA (see methods) has a bigger impact on the estimates of biodiversity. All countries and continent reach plateau in the filtered version of saMBA, suggesting that our work characterised all common taxa present in the gut microbiomes of South American individuals (Supplementary Figure 5D, 5E and 5F). However, when analysing all samples and taxa available in saMBA (Supplementary Figure 5A, 5B and 5C), it can be noted that although the identification of new unique taxa tends to decrease as more samples are inspected, the examination of novel samples still adds yet unobserved taxa. This tendency is observed even after analysing ~96% of the total samples included in saMBA

(Supplementary Figure 5A, 5B and 5C). These results suggest that although saMBA provides the most extensive characterization of gut microbiomes from South America, new samples taken across the region could still identify yet uncharacterised taxa.

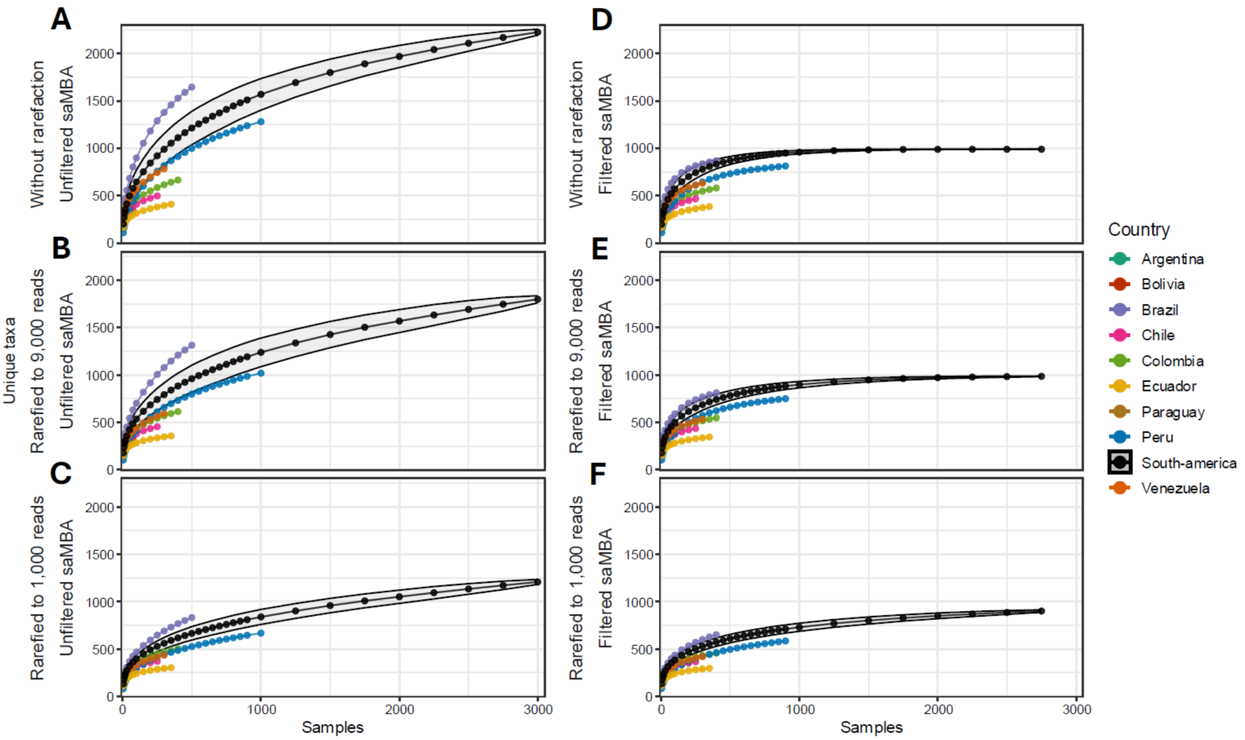

Supplementary Figure 5: Diversity estimates across the region are not altered by uneven sampling depths among countries. (A) Unique taxa identified when subsampling an increasing number of samples from each country. Each dot represents the mean value of 1,000 calculations of the number of unique taxa identified when subsampling at varying depths (i.e., number of samples). Each colour is a country. They grey area around the continental estimate (black line) represents the SD. (B) Same plot as described for A. This time, samples were rarefied to 9,000 reads before estimating the number of unique taxa for each iteration. (C) Same plot as described for A. This time, samples were rarefied to 1,000 reads before estimating the number of unique taxa for each iteration. (D) Same plot as A, after filtering taxa and samples as described in the methods. (E) Same plot as B, after filtering taxa and samples as described in the methods. (F) Same plot as C, after filtering taxa and samples as described in the methods. Note that panels A and D are the same as right and left plots in Figure 3A, respectively.

Non-diseased individuals living in non-industrialised settings across the region show a yet unveiled gut microbial biodiversity

We recognise the heterogeneity of lifestyles within South American countries. For instance, in Brazil, cities like Sao Paulo have around 44 million habitants, whereas its Amazonian regions are inhabited by small groups of people with limited contact with the industrialized world. Thus, we conducted a subsampling simulation analysis (see methods) using eight projects analysing gut microbiomes of individuals living in non-industrialized settings. We further characterised projects based on whether they included individuals with either infectious diseases or diarrhoea, or without them, as the limited metadata of some projects didn't allow us to classify at the level of individual samples. Three studies profiled subjects with diseases or diarrhoea, and the other five included individuals without any reported disease. We performed this analysis using the saMBA dataset before and after removing rare taxa and low-quality samples.

Our results suggest that saMBA characterises all common taxa present in the gut microbiomes of healthy individuals living in non-industrialised settings (Supplementary Figure 6A). However, when analysing all samples and taxa available in saMBA (Supplementary Figure 6B), it can be noted that the examination of novel samples still adds yet unobserved taxa. Our estimations of the biodiversity of this group show that when 500 samples from non-industrial healthy individuals are profiled (Supplementary Figure 6B, green line), 1,113 unique taxa are identified. This is a particularly high value considering than 1,214 unique taxa were found when profiling a set of 500 samples regardless of their industrialization status (Supplementary Figure 5A and Figure 3A, right plot). Our results thus suggest the existence of a yet unveiled gut microbiome biodiversity in South Americans living in non-industrialised contexts, specifically in those not suffering from diseases. Importantly, as control subjects were included in some projects profiling the microbiome of individuals with disease, it is likely that the biodiversity in such group is overestimated (Supplementary Figure 6A and 6B).

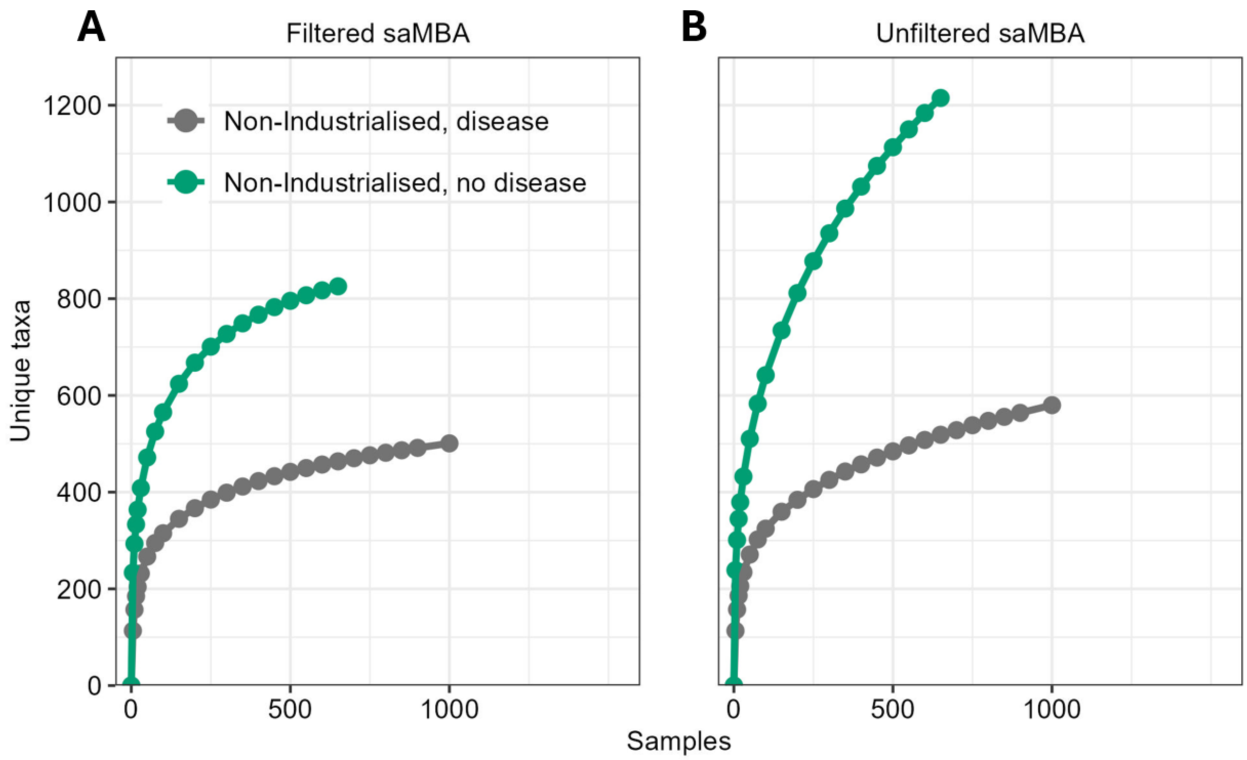

Supplementary Figure 6: Gut microbiome biodiversity estimations in subjects living in non-industrialized regions. (A) Unique taxa identified when subsampling at an increasing number of samples obtained from South Americans living in non-industrialised settings. Each dot represents the mean value of 1,000 calculations of the number of novel taxa identified when subsampling at varying depths (i.e., number of samples). The saMBA dataset after rare taxa removal was used. A total of 1,072 samples come from studies including diseased individuals, and 659 samples from studies without diseased subjects. (B) Same as A but using the saMBA dataset without removing rare taxa. A total of 1,114 samples come from studies including diseased individuals, and 665 samples from studies without diseased subjects. Green represents the biodiversity estimates using samples taken from individuals living in non-industrialised settings without disease, whereas Grey represents the same for individuals living in non-industrialised settings with infectious disease or diarrhoea.

Higher biodiversity observed in gut microbiome samples from Central America and the Caribbean compared to South America may be explained by deeper sequencing efforts.

When comparing the number of observed genera (Figure 2C) and Shannon diversity (Figure 2D) across world regions, Central America and the Caribbean showed the highest value for both alpha diversity indices. A closer inspection revealed that only two studies were conducted in this region (PRJNA397396 and PRJNA541332), both profiling the gut microbiome of adults. Furthermore, the density distribution of sequencing depths across samples (Supplementary Figure 7) showed that Central America and the Caribbean (from the HMC) peaks at a higher sequencing depth than South America (from saMBA). Although not conclusive, these observations suggest that the high biodiversity observed in Central America and the Caribbean may be due to the combination of these factors: all samples included were collected from adult subjects, and they were sequenced at higher depths when compared to the next most biodiverse region, South America (from saMBA).

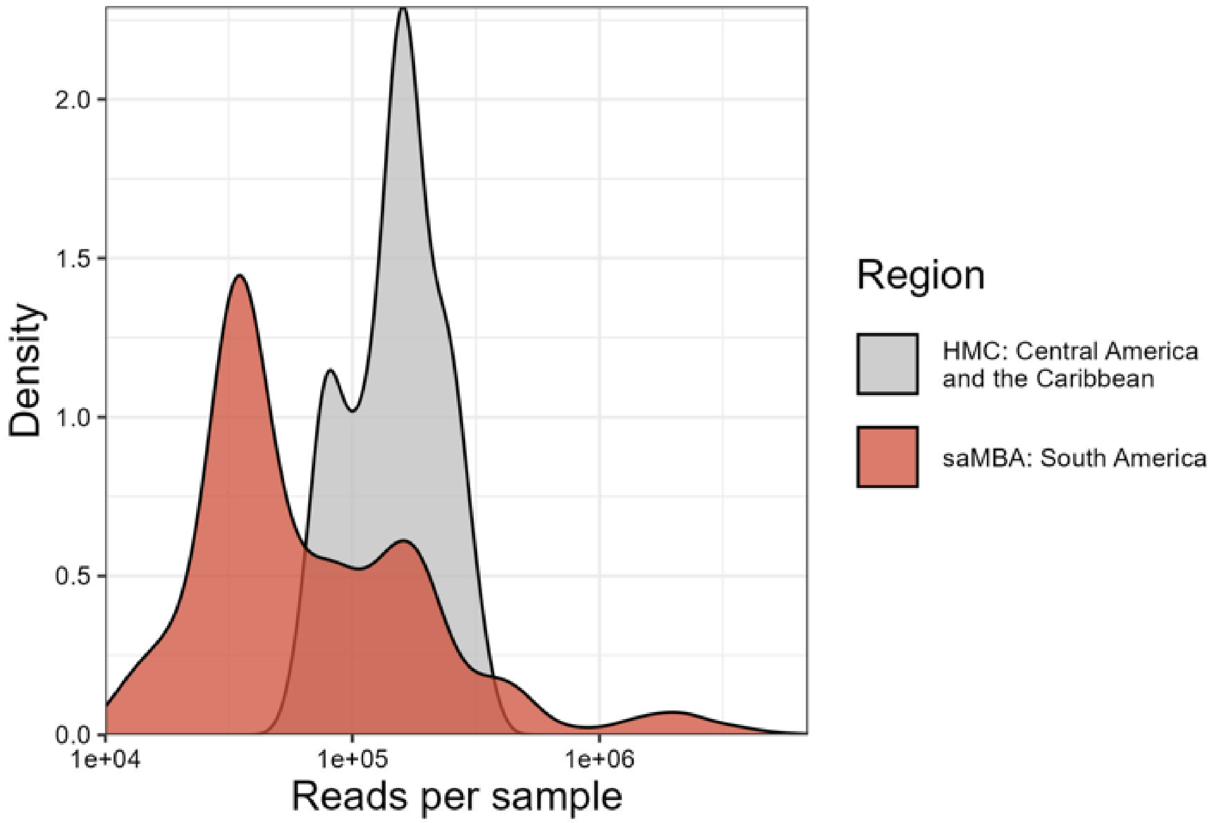

Supplementary Figure 7: Density distribution of reads per sample in South America and Central America and the Caribbean. Two world regions are included: Central America and the Caribbean, as reported in the HMC (in grey) and South America, as reported in saMBA (in red).

Supplementary references

1. Abdill, R. J. *et al.* Integration of 168,000 samples reveals global patterns of the human gut microbiome. *Cell* **188**, 1100-1118.e17 (2025).
