# Supplementary material for: The South American MicroBiome Archive (saMBA): enriching the microbiome field by studying neglected populations
Source: Nat Commun. 2025 Aug 9;16:7371. doi: 10.1038/s41467-025-62601-4 (PMC12335589; doi:10.1038/s41467-025-62601-4)
Supplement: Supplementary file 2 — Description of Additional Supplementary Files [file 41467_2025_62601_MOESM2_ESM.pdf]

File Name: Supplementary Data 1
Description: List and metadata of projects included in saMBA

File Name: Supplementary Data 2
Description: Number of projects screened and included in saMBA
