# Supplementary material for: The South American MicroBiome Archive (saMBA): enriching the microbiome field by studying neglected populations
Source: Nat Commun. 2025 Aug 9;16:7371. doi: 10.1038/s41467-025-62601-4 (PMC12335589; doi:10.1038/s41467-025-62601-4)
Supplement: Supplementary file 4 — Reporting Summary [file 41467_2025_62601_MOESM4_ESM.pdf]

nature portfolio

Corresponding author(s): Benjamin Valderrama
John F. Cryan

Last updated by author(s): Jul 14, 2025

Reporting Summary

Nature Portfolio wishes to improve the reproducibility of the work that we publish. This form provides structure for consistency and transparency in reporting. For further information on Nature Portfolio policies, see our Editorial Policies and the Editorial Policy Checklist.

Statistics

For all statistical analyses, confirm that the following items are present in the figure legend, table legend, main text, or Methods section.

n/a	Confirmed	
☐	☒	The exact sample size (*n*) for each experimental group/condition, given as a discrete number and unit of measurement
☐	☒	A statement on whether measurements were taken from distinct samples or whether the same sample was measured repeatedly
☐	☒	The statistical test(s) used AND whether they are one- or two-sided<br>*Only common tests should be described solely by name; describe more complex techniques in the Methods section.*
☐	☒	A description of all covariates tested
☐	☒	A description of any assumptions or corrections, such as tests of normality and adjustment for multiple comparisons
☐	☒	A full description of the statistical parameters including central tendency (e.g. means) or other basic estimates (e.g. regression coefficient) AND variation (e.g. standard deviation) or associated estimates of uncertainty (e.g. confidence intervals)
☐	☒	For null hypothesis testing, the test statistic (e.g. *F*, *t*, *r*) with confidence intervals, effect sizes, degrees of freedom and *P* value noted<br>*Give P values as exact values whenever suitable.*
☒	☐	For Bayesian analysis, information on the choice of priors and Markov chain Monte Carlo settings
☒	☐	For hierarchical and complex designs, identification of the appropriate level for tests and full reporting of outcomes
☐	☒	Estimates of effect sizes (e.g. Cohen's *d*, Pearson's *r*), indicating how they were calculated

Our web collection on statistics for biologists contains articles on many of the points above.

Software and code

Policy information about availability of computer code

Data collection	We generated custom code used to collect data. All code, version of the softwares and documentation about its use can be found in the following github repository and its associated github wiki page: https://github.com/Benjamin-Valderrama/saMBA-pipeline
Data analysis	All the code used to generate the analysis is publicly available and can be found in the following github repository: https://github.com/Benjamin-Valderrama/saMBA-article. Details about the specific software and versions used is available in the methods section of the manuscript.

For manuscripts utilizing custom algorithms or software that are central to the research but not yet described in published literature, software must be made available to editors and reviewers. We strongly encourage code deposition in a community repository (e.g. GitHub). See the Nature Portfolio guidelines for submitting code & software for further information.

Data

Policy information about availability of data

All manuscripts must include a data availability statement. This statement should provide the following information, where applicable:

- Accession codes, unique identifiers, or web links for publicly available datasets
- A description of any restrictions on data availability
- For clinical datasets or third party data, please ensure that the statement adheres to our policy

Provide your data availability statement here.

Research involving human participants, their data, or biological material

Policy information about studies with human participants or human data. See also policy information about sex, gender (identity/presentation), and sexual orientation and race, ethnicity and racism.

Reporting on sex and gender	Sex of the participants providing samples couldn't be identified for all studies. Therefore, our study didn't account for potential sex differences. For a clearer explanation of criteria and procedure to collect data we encourage readers to consult the methods section in the original publication.
Reporting on race, ethnicity, or other socially relevant groupings	The ethnic component relevant to this study is whether participant lived in an industrialized or non-industrialized setting. Ethnicity of the participants providing samples couldn't be identified for all studies. However, some studies presented enough metadata information to allow clear identification. In such cases, ethnicity information was included as a grouping factor, as reported in Supplementary figure 6. For a clearer explanation of criteria and procedure to collect data we encourage readers to consult the methods section in the original publication.
Population characteristics	We collected 16s amplicon datasets from human populations living in South American countries. We include all such studies, irrespective of their particular inclusion criteria, experimental design or potential diseases suffered by the studied populations.
Recruitment	For a clear explanation of criteria and procedures for recruitment and data collection we encourage to consult the original publication that produced the data we re-analysed.
Ethics oversight	For a clear explanation of criteria and procedures for recruitment and data collection we encourage to consult the original publication that produced the data we re-analysed.

Note that full information on the approval of the study protocol must also be provided in the manuscript.

Field-specific reporting

Please select the one below that is the best fit for your research. If you are not sure, read the appropriate sections before making your selection.

☒ Life sciences ☐ Behavioural & social sciences ☐ Ecological, evolutionary & environmental sciences

For a reference copy of the document with all sections, see nature.com/documents/nr-reporting-summary-flat.pdf

Life sciences study design

All studies must disclose on these points even when the disclosure is negative.

Sample size	We collected 33 16s amplicon sequencing datasets from studies profiling human gut microbiomes of populations living in south america. 3,382 samples were identified, downloaded and processed under an unified pipeline. After quality control, a total of 2,971 samples were included in the final saMBA resource. To the best of our knowledge, this is the largest collection of human microbiomes from south american subjects ever created.
Data exclusions	Individual samples were examined and quality-filtered, as reported in the HMC 8, where taxa with low number of reads across samples, and those present in a low number of samples were filtered out (50% and 14% removed on each step, respectively). Here, we aimed to remove similar proportions of taxa on each step. First, 139 samples with less than 10,000 non-chimeric reads were discarded. Then, 1123 taxonomic entries (50%) were removed as they had less than 80 reads across samples. We then removed other 133 taxa (12%), as they were present in less than 3 samples. After removing rare taxa, we aimed to remove samples with less than 10,000 remaining reads, but none were discarded. Finally, 58 samples with more than 10% of reads unclassified at the phylum level were also discarded. Therefore, the filtered count table contains 2,913 samples and 990 taxonomic identifiers.
Replication	During the development of saMBA the resource had to be created several times starting from data download to ensure the correct flow of the softwares used. Additionally, the demo run included in the documentation has been tested in two independent ubuntu machines without errors.
Randomization	Not applicable
Blinding	Not applicable

Reporting for specific materials, systems and methods

We require information from authors about some types of materials, experimental systems and methods used in many studies. Here, indicate whether each material, system or method listed is relevant to your study. If you are not sure if a list item applies to your research, read the appropriate section before selecting a response.

Materials & experimental systems

n/a	Involved in the study
☒	☐ Antibodies
☒	☐ Eukaryotic cell lines
☒	☐ Palaeontology and archaeology
☒	☐ Animals and other organisms
☒	☐ Clinical data
☒	☐ Dual use research of concern
☒	☐ Plants

Methods

n/a	Involved in the study
☒	☐ ChIP-seq
☒	☐ Flow cytometry
☒	☐ MRI-based neuroimaging

Plants

Seed stocks	*Report on the source of all seed stocks or other plant material used. If applicable, state the seed stock centre and catalogue number. If plant specimens were collected from the field, describe the collection location, date and sampling procedures.*
Novel plant genotypes	*Describe the methods by which all novel plant genotypes were produced. This includes those generated by transgenic approaches, gene editing, chemical/radiation-based mutagenesis and hybridization. For transgenic lines, describe the transformation method, the number of independent lines analyzed and the generation upon which experiments were performed. For gene-edited lines, describe the editor used, the endogenous sequence targeted for editing, the targeting guide RNA sequence (if applicable) and how the editor was applied.*
Authentication	*Describe any authentication procedures for each seed stock used or novel genotype generated. Describe any experiments used to assess the effect of a mutation and, where applicable, how potential secondary effects (e.g. second site T-DNA insertions, mosiacism, off-target gene editing) were examined.*
